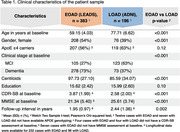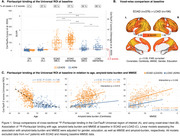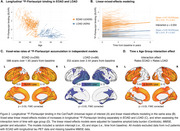# Tau PET load in early‐ and late‐onset Alzheimer's disease: A cross‐sectional and longitudinal comparison of the LEADS and ADNI cohorts

**DOI:** 10.1002/alz70856_104867

**Published:** 2026-01-07

**Authors:** Konstantinos Chiotis, Ganna Blazhenets, Daniel R. Schonhaut, Julien Lagarde, David N. Soleimani‐Meigooni, Piyush Maiti, Jiaxiuxiu Zhang, Ranjani Shankar, Alinda Amuiri, Salma Rocha, Dustin B. Hammers, Ani Eloyan, Robert A. Koeppe, Maria C. Carrillo, Brad Dickerson, Liana G. Apostolova, Renaud La Joie, Gil D. Rabinovici, the Longitudinal Early‐Onset Alzheimer's Disease Study Consortium and the Alzheimer's Disease Neuroimaging Initiative

**Affiliations:** ^1^ Memory and Aging Center, Weill Institute for Neurosciences, University of California San Francisco, San Francisco, CA, USA; ^2^ Indiana University School of Medicine, Indianapolis, IN, USA; ^3^ Department of Biostatistics, Brown University, Providence, RI, USA; ^4^ University of Michigan, Ann Arbor, MI, USA; ^5^ Alzheimer's Association, Chicago, IL, USA; ^6^ Department of Neurology, Massachusetts General Hospital and Harvard Medical School, Boston, MA, USA; ^7^ Department of Medical and Molecular Genetics, Indiana University School of Medicine, Indianapolis, IN, USA; ^8^ Department of Neurology, Indiana University School of Medicine, Indianapolis, IN, USA; ^9^ Department of Radiology and Imaging Sciences, Center for Neuroimaging, Indiana University School of Medicine, Indianapolis, IN, USA; ^10^ Department of Radiology and Biomedical Imaging, University of California San Francisco, San Francisco, CA, USA

## Abstract

**Background:**

We aimed to assess differences in baseline and longitudinal tau PET tracer binding between early‐onset Alzheimer's disease (EOAD) and late‐onset Alzheimer's disease (LOAD) in the LEADS and ADNI cohorts, respectively.

**Method:**

We analyzed amyloid‐beta PET‐positive, cognitively impaired participants from the LEADS (EOAD; *n* = 383) and ADNI (LOAD; *n* = 196) cohorts with available ^18^F‐Flortaucipir tau PET data (Table 1). A subset had longitudinal ^18^F‐Flortaucipir PET data from LEADS (*n* = 232) and ADNI (*n* = 94) with average follow‐up intervals of 1.95 and 2.44 years, respectively. All ^18^F‐Flortaucipir PET scans were processed using the CenTauR pipeline. Cognitively normal participants from LEADS (*n* = 94) and ADNI (*n* = 421) with baseline ^18^F‐Flortaucipir and amyloid‐beta PET scans were also analyzed for comparison. We performed EOAD vs. LOAD comparisons using multivariate linear and linear mixed‐effects models for cross‐sectional and longitudinal analyses, respectively.

**Result:**

Baseline comparisons revealed large effect‐size, significant differences in ^18^F‐Flortaucipir binding between EOAD and LOAD (Figure 1). EOAD participants had higher ^18^F‐Flortaucipir levels in widespread neocortical regions compared to LOAD, after adjusting for covariates. In both groups, tau load was negatively associated with age. In EOAD, a significantly steeper slope was found in the association between amyloid‐beta and ^18^F‐Flortaucipir load, as well as between cognitive scores and ^18^F‐Flortaucipir load. Longitudinally, EOAD participants exhibited a faster increase in ^18^F‐Flortaucipir binding than LOAD, predominantly in frontal and occipital areas (Figure 2), with both groups showing an inverse linear relationship between ^18^F‐Flortaucipir accumulation rates and age.

**Conclusion:**

EOAD patients demonstrate significantly higher tau loads, broader neuroanatomical involvement and faster tau accumulation over time compared to LOAD, independent of disease stage. These findings suggest that earlier age‐of‐onset in AD is linked to a more aggressive tauopathy. The early, extensive tau spread in symptomatic EOAD, even at an early clinical stage, may also limit the efficacy of anti‐amyloid‐beta therapies in this population.